# Study protocol for evaluating Six Building Blocks for opioid management implementation in primary care practices

**DOI:** 10.1186/s43058-020-00008-6

**Published:** 2020-02-26

**Authors:** Sarah J. Shoemaker-Hunt, Leigh Evans, Holly Swan, Olivia Bacon, Brooke Ike, Laura-Mae Baldwin, Michael L. Parchman

**Affiliations:** 1 Division of Health and Environment, Abt Associates, Inc., Cambridge, USA; 2 Department of Family Medicine, University of Washington, Seattle, USA; 3 Kaiser Permanente Washington Health Research Institute, Seattle, USA

**Keywords:** Opioid management, Evaluation design, Practice redesign, Primary care, Quality improvement, Chronic pain management, Prescribing practices

## Abstract

**Background:**

The Six Building Blocks for improving opioid management (6BBs) is a program for improving the management of patients in primary care practices who are on long-term opioid therapy for chronic pain. The 6BBs include building leadership and consensus; aligning policies, patient agreements, and workflows; tracking and monitoring patient care; conducting planned, patient-centered visits; tailoring care for complex patients; and measuring success. The Agency for Healthcare Research and Quality funded the development of a 6BBs implementation guide: a step-by-step approach for independently implementing the 6BBs in a practice. This mixed-method study seeks to assess practices’ use of the implementation guide to implement the 6BBs and the effectiveness of 6BBs implementation on opioid management processes of care among practices using the implementation guide.

**Methods:**

Data collection is guided by the Consolidated Framework for Implementation Research, Proctor’s taxonomy of implementation outcomes, and the Centers for Disease Control and Prevention’s Guideline for Prescribing Opioids for Chronic Pain. A diverse group of health care organizations with primary care clinics across the USA will participate in the study over 15 months. Qualitative data collection will include semi-structured interviews with stakeholders at each organization at two time points, notes from routine check-in calls, and document review. These data will be used to understand practices’ motivation for participation, history with opioid management efforts, barriers and facilitators to implementation, and implementation progress. Quantitative data collection will consist of a provider and staff survey, an implementation milestones assessment, and quarterly opioid prescribing quality measures. These data will supplement our understanding of implementation progress and will allow us to assess changes over time in providers’ opioid prescribing practices, prescribing self-efficacy, challenges to providing guideline-driven care, and practices’ opioid prescribing quality measures. Qualitative data will be coded and analyzed for emergent themes. Quantitative data will be analyzed using descriptive statistics and clustered multivariate regression.

**Discussion:**

This study contributes to the knowledge of the implementation and effectiveness of a team-based approach to opioid management in primary care practices. Information gleaned from this study can be used to inform efforts to curtail opioid prescribing and assist primary care practices considering implementing the 6BBs.

Contributions to the literature

- This study will improve understanding about how to guide primary care practices in initiating, implementing, and sustaining an opioid management quality improvement effort.
- Improving opioid management can be challenging for primary care clinics to pursue. This study will identify strategies used to de-implement previous practices, change processes of care, and provide clinicians with resources for engaging in often difficult conversations with patients.
- Lessons learned from this study will be disseminated to support other primary care practices as they implement strategies to address the opioid crisis in their practices and communities.

## Background

In 2017, the number of overdose deaths involving opioids was 130 Americans per day, which was six times higher than in 1999 [1]. While the national opioid prescribing rate declined from 2012 to 2016 to 58.7 prescriptions per 100 persons (191 million prescriptions); in 16% of US counties, there are enough opioid prescriptions for every person to have one [1]. In addition, annual prescription rates for 30 days or more of opioids increased by 59% between 2006 and 2012 and have not decreased since that time. The Department of Health and Human Services (HHS) declared this a public health emergency, and HHS agencies have pursued several initiatives to address the opioid epidemic.

One of those initiatives is the Centers for Disease Control and Prevention (CDC)’s *Guideline for Prescribing Opioids for Chronic Pain*, released in March 2016. This guideline outlines several evidence-based opioid management strategies for primary care clinicians [2], who account for about half of the opioid pain relievers prescribed [3]. It has been well established that the publication of evidence-based guidelines alone is not sufficient to change care delivery, especially in diverse primary care settings with significant competing demands and limited resources [4–6]. The care delivered in these settings is influenced by more than just provider knowledge and attitude. It is also influenced by how care is organized within a clinic team. It requires a team-based approach supported by changes to clinic systems and workflows within teams to ensure that care is safe and effective [7].

**Fig. 1:**
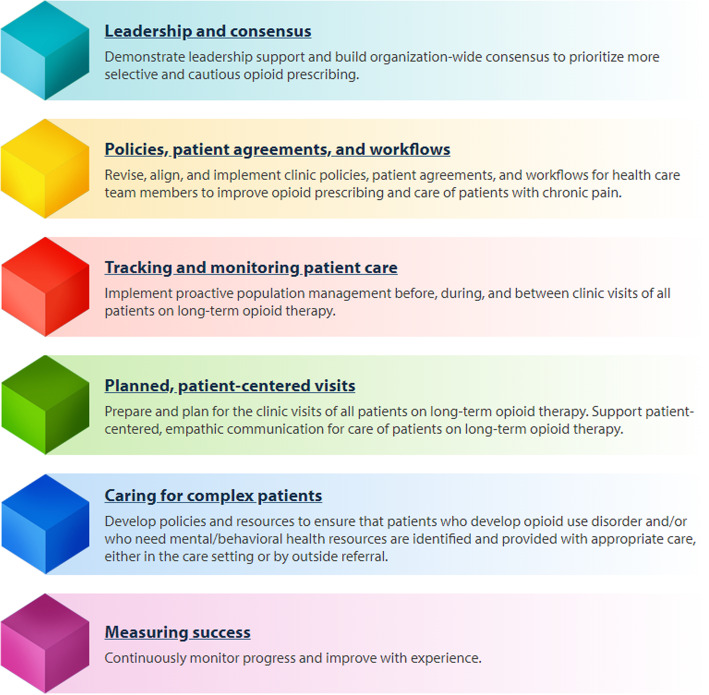
Six Building Blocks: a team-based approach to improving opioid management in primary care. Note: Graphic retrieved from https://depts.washington.edu/fammed/improvingopioidcare/6-building-blocks/

Several of the authors (MP, LMB, BI), as part of a grant from the Agency for Healthcare Research and Quality (AHRQ), conducted research on how to support primary care practices with clinic redesign to improve opioid management and provide safer care using a team-based approach. To guide clinic improvement teams and those providing external support to those teams, they developed the Six Building Blocks for team-based opioid management (6BBs) based on earlier observations of high-functioning teams in exemplar primary care practices [8]. The 6BBs (see Fig. 1) include (1) leadership support; (2) revision and alignment of clinic policies, patient agreements, and workflows; (3) tracking and monitoring the population of patients using long-term opioid therapy (LtOT); (4) planned, patient-centered visits; (5) identifying resources for complex patients; and (6) measuring success. An evaluation of the 6BBs found significant declines in both the total number of patients receiving opioids for their chronic pain and the proportion of patients on higher dose opioids [9].

The 6BBs program was developed as a quality improvement (QI) approach to be implemented with external support from a practice facilitator [10]. Such external support systems are often not available to primary care practices. The purpose of this study is to understand the feasibility of a primary care clinical organization independently implementing improvements to opioid management using a 6BBs “how-to-guide.”

Given AHRQ’s mission to address patient safety threats with health services research and its charge to support research on improving primary care and practice transformation, along with the HHS Secretary’s call to address the opioid crisis with evidence-based resources, AHRQ funded this study to examine primary care practices’ implementation of practice redesign around opioid management using the 6BBs self-service guide, and its effectiveness in transforming their care practices and opioid prescribing, all without the help of a practice facilitator. 

While there have been some studies of opioid stewardship in primary care settings [11–16], most have been within one health care system and have not examined differences across systems and practices. Additionally, only a few of these studies examined implementation explicitly, which is a critical component of putting evidence-based interventions into practice. This study examines the implementation and effectiveness of a specific “self-guided” model for opioid management QI across practices of different sizes, populations served, and geographies.

## Methods

### Study objectives and design

The implementation objective of this study is to:

- Understand the adoption and implementation of the 6BBs among participating health care organizations.

The effectiveness objective of this study is to:

- Assess the effectiveness of 6BBs implementation on opioid management practices and processes of care.

This study employs a hybrid type III implementation-effectiveness design [17]. It uses mixed methods, collecting both quantitative and qualitative data from primary and secondary data sources. Data will be collected prospectively at multiple time points over the 24-month study period. The first 6 months of the study were dedicated to developing, testing, and refining the 6BBs how-to-guide (Clinic Implementation Guide); months 7–21 will involve 6BBs implementation, including the use of the Clinic Implementation Guide and data collection activities; and the last 4 months will entail data analysis, final modifications to the 6BBs Clinic Implementation Guide for widespread dissemination, and reporting of findings.

### Conceptual frameworks

The Consolidated Framework for Implementation Research (CFIR) [18] and Proctor’s taxonomy of implementation outcomes [19] guide the implementation component of this study. CFIR describes factors important to implementation in terms of intervention characteristics, outer setting (external factors influencing implementation), inner setting (internal factors influencing implementation), characteristics of those involved in implementation, and the implementation process. Proctor’s taxonomy informs the outcomes we can expect to see throughout the course of sites using the Clinic Implementation Guide and implementing the 6BBs; in this study, we will primarily focus on the implementation outcomes of acceptability, adoption, feasibility, penetration, and sustainability.

This study’s effectiveness component is examined, in part, by the clinical quality improvement (QI) opioid measures developed by the authors (SS, HS) and published in the CDC’s Quality Improvement and Care Coordination: Guideline for Prescribing Opioids for Chronic Pain [20]. Four of these opioid prescribing QI measures will be used to assess effectiveness of 6BBs implementation: the proportion of patients with chronic non-cancer pain using long-term opioid therapy who (1) are prescribed greater than 90 morphine milligram equivalents (MMEs) per day, (2) are co-prescribed a benzodiazepine, (3) had the prescription drug monitoring program (PDMP) checked, and (4) had a urine drug screen.

### Sample and recruitment

We recruited a diverse group of health care organizations with primary care clinics to participate in this study. A notice about the study and the opportunity to participate was posted on AHRQ’s website. Additionally, organizations were notified through AHRQ’s Prevention and Chronic Care email list, Primary Care Practice-Based Research Networks email update, and Practice Facilitation email update, and through their connections with the study team. Interested organizations sent a request for more information to a member of the study team, who then compiled information about the organization and its clinics from publicly available information on the organization’s website, including number of clinics, location, patient population, and relevant academic affiliation. Brief screening calls were held with interested organizations to provide them with more information about the study and explain expectations for participation and to gather information about the organization’s motivation for participation, past experience with similar QI initiatives, goals and expectations, and use of electronic health record (EHR) system for QI and expected vendor changes in the near future. These calls helped forge relationships with potential participating organizations and assess study alignment with their organizational goals—two strategies identified as important for facilitating health care QI initiative recruitment [21, 22].

We conducted screening calls with 30 health care organizations. To date, we selected a purposive sample of 11 organizations across 9 US states to invite to participate in the study (see Table 1), which currently include 40 associated primary care clinics. Participating organizations vary with respect to size, number of primary care clinics, patient population served, geographic location, and academic affiliation. This variation will allow us to examine and compare implementation successes and challenges across a range of organizations likely to be targeted by widespread 6BBs dissemination upon study completion.

**Table 1:** Participating health care organization characteristics

Health care organization	US census division	Location	No. of clinical sites^†^	Organization characteristics	Experience with opioid management	Opioid prescribing rate per 100 (county)*	Opioid OD death rate per 100,000 (state)**
Org. 1	Pacific	Rural	4	• Multispecialty group practice • Serves about 72,000 patients • Community hospital subsidiary	• Currently participating in Medicaid transformation project on 6BBs	90.4	9.4
Org. 2	Pacific	Urban	13	• Federally Qualified Health Center (FQHC) • Patient-centered medical home (PCMH) • Community-based nonprofit organization • Serves about 30,000 patients; over 50% of patients have Medicaid coverage	• Familiar with 6BBs • Has a system-wide, cross discipline policy including prescribing guidelines, refill policy, and MAT referrals/treatment • Interested in self-service approach to tweak what is already in place	56.8	9.7
Org. 3	Pacific	Urban	1	• University affiliated • Part of a health system • 50% of patients are racial/ethnic minorities	• Has done a lot of work but not streamlined • Has been developing opioid policy	27.1	5.3
Org. 4	Mountain	Urban	1	• Privately owned primary care practice • PCMH • Recognized as an exemplar practice by other initiatives	• Has conducted work around opioid management for years, including efforts to stratify patients according to opioid abuse risk	47.2	9.5
Org. 5	Mountain	Rural	4	• FQHC • PCMH • High percentage of patients on Medicare/Medicaid • 22 total clinic sites in the system	• Seeking alternative pain management practices for providers • Due to limited availability of pain management specialists, many primary care providers prescribe	111.8	11.4
Org. 6	East North Central (Midwest)	Urban	6	• FQHC • University affiliated • 80% of patients have Medicaid coverage	• Does not do a lot of new prescribing of opioids but faces challenges managing inherited patients on opioids	37.2	15.3
Org. 7	East North Central (Midwest)	Urban	1	• Hospital-owned, family medicine residency program • PCMH • Part of a health system	• Has worked on implementing components of the 6BBs including workflows, policies, and patient agreements	61.8	32.9
Org. 8	East North Central (Midwest)	Urban	1	• Residency clinic • University affiliated	• Has guidelines from hospital and some of the pieces already in place in the residency program • Current opioid education is focused on risk management	50.1	32.9
Org. 9	East South Central	Rural	6	• Regional hospital with outreach clinics. 16 total clinic sites in the system • PCMH	• Started an opioid stewardship committee and wants to include best practices in building this program • Has worked on policies and patient agreements and now wants to focus on tracking/monitoring.	96.9	23.6
Org. 10	Mid-Atlantic	Rural	2	• Graduate medical education safety-net consortium • Serves about 17,500 patients • PCMH • Part of a health system; 9 total clinic sites in system	• Has conducted prior work on opioid prescribing and aims to improve further	97.3	18.5
Org. 11	Mid-Atlantic	Urban	1	• Hospital-owned, family medicine residency clinic • Serves about 22,000 patients • University affiliated • Part of a health system	• Has worked on implementing components of the 6BBs for several years, but sees an opportunity to improve	42.7	15.1

^†^This is the number of clinical sites in the organization participating in implementing the Six Building Blocks. This is not necessarily the total number of clinical sites in the health system/organization

*County-level rate of retail opioid prescriptions dispensed per 100 people. Obtained from Centers for Disease Control and Prevention, US County Prescribing Rates, 2017, available at https://www.cdc.gov/drugoverdose/maps/rxcounty2017.html; national average prescribing rate in 2017 was 58.7 prescriptions per 100 persons

**Opioid-related overdose deaths per 100,000. Obtained from the National Institute of Drug Abuse, State Opioid Related Overdose Death Rates, 2016, available at https://www.drugabuse.gov/drugs-abuse/opioids/opioid-summaries-by-state; national average opioid-related overdose deaths in 2016 was 13.3 deaths per 100,000 persons

### Data collection and measures

#### Qualitative data

Qualitative data sources will heavily inform the implementation objective of this study and include staff interviews; notes from orientation and quarterly calls; emails from sites to the study team; completed Clinic Implementation Guide materials; and practice documents, such as policies and workflows. We will use these sources to capture data about organizations’ implementation progress, extent of use of the Clinic Implementation Guide, and barriers and facilitators organizations encounter while using the Clinic Implementation Guide and implementing the 6BBs (see Table 2).

**Table 2:** Data sources

Source	Unit of observation	Type	Domains/content	Frequency
Semi-structured staff interviews	QI leads, clinical champions, key staff involved in implementation	Qualitative	Adaptability, complexity, design quality and packaging, execution* Barriers and facilitators to implementation Adoption, acceptability, feasibility, appropriateness, sustainability** Lessons learned	At study startup, midway through, and end
Call notes/emails	Health care organizations	Qualitative	Barriers and facilitators to implementation Adaptability, acceptability, feasibility**	Ongoing
Practice documents	Health care organizations	Qualitative	Intermediate outcomes and care processes (6BBs): Opioid prescribing policies and procedures, clinical workflows, dashboards, patient registries, patient agreements, training and education offerings, opioid tracking and monitoring reports, care plan templates, patient educational materials, screening tools related to opioid management, referral processes between primary care and behavioral health and/or pain specialists	Ongoing
Clinical staff survey	Clinical staff: primary care providers, behavioral health providers, nurses, social workers, medical assistants	Quantitative	Penetration** Engagement in implementation Prescribing self-efficacy (prescribers only) Burnout Adaptive reserve***	At study startup and 12 months later
6BBs Milestones worksheet	Health care organizations	Quantitative	Adoption and fidelity**	At study startup, midway through, and end
Opioid quality improvement measures	Clinics within health care organizations	Quantitative	Effectiveness measures (CDC Guideline): • Proportion of patients on long-term opioid therapy prescribed ≥ 90 MMEs per day • Proportion of patients on long-term opioid therapy co-prescribed a benzodiazepine • Proportion of patients with a new opioid prescription for chronic pain with documentation that a prescription drug monitoring program was checked prior to prescribing • Proportion of patients with a new opioid prescription for chronic pain with documentation that a urine drug test was performed prior to prescribing	Quarterly for study duration

*The Consolidated Framework for Implementation Research. 2019. Available from https://cfirguide.org/constructs/

**Proctor et al. [19]

***Jaén et al. [23]

##### Staff interviews

The research team will conduct semi-structured interviews with the QI lead and four additional staff members involved in implementation (*n* = 5) at each organization at two time points. Interviewed clinical staff members will include the designated clinical champion and a primary care provider, and up to 2 others such as the Medical Director, pharmacist, data analyst, office manager, refill manager, behavioral health provider, addiction specialist, Suboxone waivered clinician, or an alternative therapy provider. Interviews will be conducted once toward the beginning of their QI effort and again at the end of the study. The initial interview will focus on plans for implementation and encountered barriers, while the second interview will focus on sustainability plans and lessons learned.

The content of the QI lead and staff interview guides will be based on CFIR constructs and will capture each organization’s plans for and progress with using the 6BBs Clinic Implementation Guide to implement improvements in opioid management, challenges and successes related to guide utilization and quality improvement implementation, perceived effect of using the 6BBs Clinic Implementation Guide on patient care and organizational processes, and plans for sustainability. Interviews will last approximately 1 h, be conducted by phone, and be audio recorded with participant consent. Participants will be offered monetary incentives.

##### Orientation and quarterly call notes and email text data

An orientation meeting will be held by web-conference for all participating organizations to introduce and orient them to the 6BBs Clinic Implementation Guide and launch implementation. Additionally, the study team will hold quarterly calls with each participating site to receive an update on implementation progress, struggles, and challenges. Notes will be taken during the orientation and quarterly calls. Organizations will also have the ability to email the study team with questions or comments about implementation and using the Clinic Implementation Guide. Call notes and email text data will be used as data sources to capture barriers and facilitators to adoption and use of the 6BBs Clinic Implementation Guide to implement opioid management improvements.

##### Practice documents

We will collect from each participating organization and clinic documentation of changes implemented through using the 6BBs Clinic Implementation Guide, such as opioid prescribing policies, written workflows, patient agreements, screenshots of opioid prescribing dashboards and/or registries, de-identified tracking and monitoring reports for patients using long-term opioid therapy, training and education offerings (past, present, future), and patient education materials discussing risks and benefits of long-term opioid use. Baseline documents will be collected at study start, and clinics and organizations will be asked to submit all updated documents throughout the course of the study to track 6BBs implementation progress.

#### Quantitative data

##### Clinical staff survey

We will conduct an electronic survey of clinical staff at participating organizations at the beginning of implementation and again at the end of the study to capture information about opioid prescribing practices and opioid management procedures, derived from the CDC Guideline for Prescribing Opioids for Chronic Pain, and engagement in 6BBs implementation, guided by CFIR. A link to the survey will be emailed to clinical staff identified by the QI lead as involved in opioid prescribing and/or management within the clinic. The respondent population will include primary care providers, behavioral health providers, nurses, pharmacists, social workers, and/or medical assistants. The survey will also assess adaptive reserve, defined as perceived practice attributes, including leadership, culture, and communication, that indicate successful organizational change and signal improvements in patient care [23, 24]. Adaptive reserve will be measured using the Practice Adaptive Reserve Scale [23]. Clinical staff who prescribe opioids will also be asked about their self-efficacy around opioid management and perceived burnout using a validated single-item measure of burnout [25].

##### 6BBs Milestones worksheet

The 6BBs Milestones worksheet outlines key implementation milestones to be reached within each of the six areas addressed by the 6BBs. The proportion of milestones reached for each area will serve as a measure of implementation progress. All organizations (*n* = 11) will be asked to complete this worksheet at the start of, midway through, and at the end of the study.

##### Quarterly reports of aggregate QI measures

Each organization (*n* = 11) will submit quarterly aggregate reports to the study team on the four quality improvement measures derived from the CDC Guideline for Prescribing Opioids for Chronic Pain: (1) the percentage of patients using long-term opioid therapy who are taking 90 MMEs or more per day, (2) the percentage of patients using long-term opioid therapy who received a prescription for a benzodiazepine, (3) the percentage of patients with a new opioid prescription for chronic pain with documentation that the state Prescription Drug Monitoring Program (PDMP) was checked prior to prescribing, and (4) the percentage of patients with a new opioid prescription for chronic pain with documentation that a urine drug test was performed prior to prescribing. These measures track organization-level opioid prescribing practices and have been recognized as valid measures of opioid management and used in other studies [11, 15, 16].

### Data analysis

#### Analytic plan

Using the complementarity function as described in the taxonomy of mixed methods designs in implementation research [26], quantitative findings will be interpreted in the context of implementation findings for each organization as gathered through qualitative analysis. We will also use a comparative case study approach [27] to compare the similarities and differences across all organizations in the study in their ability to use the 6BBs Clinic Implementation Guide to implement improvements in each of the 6BBs areas and opioid management.

Our analytic plan is guided by our objectives. Objective 1 (implementation) includes an assessment of each organization’s implementation and use of the 6BBs Clinic Implementation Guide and the implementation of improvements using the 6BBs overall. Objective 2 (effectiveness) includes assessment of the effectiveness of 6BBs implementation on processes of care and outcomes.

#### Approach for objective 1 (implementation): to understand adoption and implementation of the 6BBs among participating health care organizations

Objective 1 is to understand the barriers and facilitators to using the 6BBs Clinic Implementation Guide to implement improvements in each of the 6BBs (i.e., leadership and consensus; policies, patient agreements, and workflows; tracking and monitoring patient care; planned, patient-centered visits; caring for complex patients; measuring success). Data to address this aim will come from five main sources: (1) practice documents, (2) orientation and quarterly call notes and email text data, (3) QI lead and staff interviews, and (4) the 6BBs Milestones worksheet.

These qualitative data will be used to understand the barriers and facilitators to implement the 6BBs (see Table 2). These data will be imported into NVivo and coded for themes around barriers and facilitators to implementation of using the 6BBs Clinic Implementation Guide to implement improvements to opioid management. Initial deductive coding will be guided by the CFIR domains, Powell and colleagues’ implementation strategies [28], and each component of the 6BBs. Coding will also be inductively updated through thematic analyses, with a particular focus on staff experiences around using the Clinic Implementation Guide and its utility for facilitating 6BBs uptake, as well as suggested changes to the Clinic Implementation Guide to improve its usefulness related to each component.

Practice documents and the 6BBs Milestones worksheet will be reviewed to determine organizations’ progress on achieving major milestones in each area. Particularly, the proportion of milestones achieved within each area will be compared at the organizational level across the three time points at which those data are collected. Due to the small sample size of participating organizations, data will be analyzed using descriptive statistics of the proportion of milestones reached.

As a supplemental data source, barriers to opioid management indicated on the clinical staff survey will be analyzed and compared from the start to the end of the study. The number and type of barriers and facilitators reported will be summarized descriptively, including mean, median, standard deviations, and change over time both at the organizational level and pooled across all organizations.

#### Approach for objective 2 (effectiveness): to evaluate the effect of 6BBs toolkit implementation on processes of care and intermediate outcomes

We will use quantitative data from the clinical staff survey and quarterly reports of QI measures to assess the effectiveness of 6BBs implementation. The primary effectiveness outcomes from the survey are clinician prescribing practices (e.g., using the PDMP, using a registry to manage patients using long-term opioid therapy, developing a treatment agreement, discussing risks and benefits of opioid therapy with patients, and calculating daily morphine equivalent dosing). Secondary outcomes are clinician prescribing self-efficacy and reported adaptive reserve. Survey data will be exported from Survey Gizmo’s online system and imported into SAS for analysis. We will generate descriptive statistics for survey variables, including mean, median, standard deviation, and plots of distributions for continuous variables, and frequency tables and plots for categorical variables.

Survey responses will be pooled across health care organizations. We expect to receive approximately 450 clinical staff surveys at each of the two time points. A multi-level regression model will be used to determine the association between a pre-post dummy variable and change in each of the three outcomes: clinician prescribing practices, prescribing self-efficacy, and adaptive reserve. The regression model will be adjusted for respondent characteristics captured on the survey, such as clinician type and years of experience. The multi-level model will account for clustering of clinical staffing within health care organizations. A power analysis for a paired-samples *t* test assuming 80% statistical power, significance level of 0.05, and standard deviation of 1 shows the minimum detectable effect is 0.13 from pre to post. Similar or larger effect sizes have been determined for changes in providers’ opioid prescribing knowledge, attitudes, and beliefs, self-efficacy, and opioid management practices following other opioid management QI projects [14, 29].

The QI measures we will track as organizational-level outcomes are (1) the percentage of patients using long-term opioid therapy who are taking 90 MMEs or more per day, (2) the percentage of patients using long-term opioid therapy who received a prescription for a benzodiazepine, (3) the percentage of long-term opioid therapy patients with a new opioid prescription for non-cancer chronic pain with documentation that a PDMP was checked prior to prescribing, and (4) the percentage of long-term opioid therapy patients with a new opioid prescription for non-cancer chronic pain with documentation that a urine drug test was performed prior to prescribing. We will report changes in QI measures over time at the clinic level using descriptive statistics due to the small sample size of clinics expected to be enrolled in the study (*n* = 11).

## Discussion

As the use of prescription opioids continues to impact the opioid epidemic, it is critical to understand the factors that influence health care systems’ ability to implement guideline-concordant opioid management practices. The 6BBs Clinic Implementation Guide offers a structured program of strategies, grounded in evidence, designed to facilitate the implementation of complex and multi-faceted opioid management practices that align with the CDC Guideline for Prescribing Opioids for Chronic Pain [2]. Understanding the determinants of success and systematic barriers to implementing and sustaining opioid management strategies in primary care practices will enable researchers and practitioners to spread effective implementation strategies to address the opioid epidemic in the health care system.

This hybrid type III study advances implementation science by applying established frameworks and a validated instrument (i.e., adaptive reserve) for evaluating implementation to a new context: opioid management in primary care clinics. Diverse practices will be recruited for this study because primary care clinics are not controlled settings; each clinic has different complexities and contextual factors that influence the extent to which implementation will be successful. The application of the CFIR and Proctor’s model of implementation outcomes to opioid management in primary care settings provides a structure from which to systematically examine these factors. Moreover, the use of the adaptive reserve, a validated instrument for evaluating organizational factors, will inform its utility for evaluating the implementation of opioid management strategies in primary care.

### Challenges

The primary focus of this study is to understand the implementation processes, successes, and challenges for each of the participating clinics. However, as a feature of the hybrid type III design being used for this study, quantitative data will be collected to also evaluate effectiveness of using the 6BBs Clinic Implementation Guide to improve outcomes related to opioid management (e.g., the percentage of patients using long-term opioid therapy who are taking 90 MMEs or more per day). The small sample size of organizations included in the study may limit the ability to detect effectiveness; additionally, the evaluation does not incorporate randomization or a counterfactual, which limits our ability to make causal claims about the effectiveness of the 6BBs on clinical outcomes. An additional challenge that is common when conducting survey research is the potential for a low response rate on the clinician survey, which could limit the internal validity of the survey’s findings. We are addressing this challenge through our targeted recruitment strategy and electronic modality of the survey. Finally, the diversity of practices recruited for this study was intentional in order to capture a breadth of contextual factors that influence implementation; however, this diversity may also pose a challenge for drawing overarching conclusions.

### Project status

At the time of writing, the majority of participating organizations have been enrolled and we are initiating baseline data collection. Practices will be given the 6BBs Clinic Implementation Guide in the spring of 2019, at which point we will launch the main components of study data collection.

## Data Availability

Not applicable

## Abbreviations

6BBs: The Six Building Blocks for improving opioid management
AHRQ: Agency for Healthcare Research and Quality
CDC: Centers for Disease Control and Prevention
CFIR: Consolidate Framework for Implementation Research
EHR: Electronic health record
FQHC: Federally Qualified Health Center
HHS: US Department of Health and Human Services
LtOT: Long-term opioid therapy
MAT: Medication-assisted treatment
MMEs: Morphine milligram equivalents
PCMH: Patient-centered medical home
PDMP: Prescription drug monitoring program
QI: Quality improvement
